# Barley Genotypes Vary in Stomatal Responsiveness to Light and CO_2_ Conditions

**DOI:** 10.3390/plants10112533

**Published:** 2021-11-21

**Authors:** Lena Hunt, Michal Fuksa, Karel Klem, Zuzana Lhotáková, Michal Oravec, Otmar Urban, Jana Albrechtová

**Affiliations:** 1 Department of Experimental Plant Biology, Faculty of Science, Charles University, Viničná 5, 12844 Praha, Czech Republic; huntl@natur.cuni.cz (L.H.); Michal.Fuksa@gmail.com (M.F.); zuzana.lhotakova@natur.cuni.cz (Z.L.); 2 Global Change Research Institute, Czech Academy of Sciences, Bělidla 4a, 60300 Brno, Czech Republic; klem.k@czechglobe.cz (K.K.); oravec.m@czechglobe.cz (M.O.); urban.o@czechglobe.cz (O.U.)

**Keywords:** stomata, stomatal density, stomatal conductance, stomatal regulation, barley, CO_2_, light, ABA, neural network, phenolics

## Abstract

Changes in stomatal conductance and density allow plants to acclimate to changing environmental conditions. In the present paper, the influence of atmospheric CO_2_ concentration and light intensity on stomata were investigated for two barley genotypes—Barke and Bojos, differing in their sensitivity to oxidative stress and phenolic acid profiles. A novel approach for stomatal density analysis was used—a pair of convolution neural networks were developed to automatically identify and count stomata on epidermal micrographs. Stomatal density in barley was influenced by genotype, as well as by light and CO_2_ conditions. Low CO_2_ conditions resulted in increased stomatal density, although differences between ambient and elevated CO_2_ were not significant. High light intensity increased stomatal density compared to low light intensity in both barley varieties and all CO_2_ treatments. Changes in stomatal conductance were also measured alongside the accumulation of pentoses, hexoses, disaccharides, and abscisic acid detected by liquid chromatography coupled with mass spectrometry. High light increased the accumulation of all sugars and reduced abscisic acid levels. Abscisic acid was influenced by all factors—light, CO_2_, and genotype—in combination. Differences were discovered between the two barley varieties: oxidative stress sensitive Barke demonstrated higher stomatal density, but lower conductance and better water use efficiency (WUE) than oxidative stress resistant Bojos at saturating light intensity. Barke also showed greater variability between treatments in measurements of stomatal density, sugar accumulation, and abscisic levels, implying that it may be more responsive to environmental drivers influencing water relations in the plant.

## 1. Introduction

Stomata are made up of paired guard cells which regulate the aperture of a pore through changes in their turgor, allowing controlled gas exchange between the atmosphere and internal air spaces of a plant. Guard cells are flanked by subsidiary cells, which act as reservoirs of water and osmolytes that can facilitate guard cell movement [1]. Open stomata allow for gas exchange mediated by concentration gradients resulting in an influx of CO_2_, the substrate of photosynthesis, and efflux of O_2_ and H_2_O, the latter providing transpirational cooling of the plant. Closed stomata conserve water inside the plant necessary for physiological functions and act as a barrier to pollutants and pathogens. They serve a key role in plant productivity by balancing the photosynthetic CO_2_-demand with maintaining a suitable temperature and water status for the plant. Although stomata occupy only 0.3–5% of leaf epidermal surface, they account for up to 95% of all gas exchange between the plant and atmosphere [2], while cuticular transpiration makes up the remainder. The recent advances in research on the plasticity of stomatal development and regulation make stomata a key feature in eco-physiological investigations of plant adaptation and a target for increasing agricultural productivity under the limiting environmental conditions of ongoing climate change.

The number of stomata on leaves may increase or decrease according to environmental cues. Mature leaves sense environmental cues and transmit signals to determine the frequency of stomata on developing leaves [3]. Allocation of epidermal space to stomata is constrained by the cost of developing and operating stomata [4] and both an inadequate and an excessive number of stomata present significant trade-offs in plant productivity and survival [5]. The stomatal responses to light and atmospheric CO_2_ concentration ([CO_2_]) oppose each other—increasing light tends to increase stomatal density, while increasing [CO_2_] tends to decrease stomatal density [6], although exceptions exist [7,8]. Changes in atmospheric [CO_2_] have led to observable decreases in stomatal density in modern plants compared to pre-industrial herbarium samples [9] and fossilized plants [10]. However, work by Zhang et al. [11] suggests that the determination of stomatal density is species-specific, with some species influenced mainly by genetics, and others more responsive to environmental drivers. It is, therefore, useful to examine the influence of environmental cues on both the density and function of stomata among genotypes within a single species. 

In the short term, gas exchange is regulated by the aperture of the stomatal pore, a phenomenon that occurs via changes in guard cell turgor pressure mediated by complex signaling cascades. Accumulation of potassium ions, chloride, and organic ions (such as malate and sucrose) in guard cells causes water uptake from the apoplast, resulting in high turgor and stomatal opening [12]. Stomatal closure occurs when ions and solutes are released, inducing a loss of turgor as water moves out of the guard cells. Stomatal conductance (*G_S_*), a measure of the simultaneous influx of CO_2_ and efflux of water vapor occurring through stomatal pores, is an integral of stomatal density and stomatal opening. *G*_S_ is driven by photosynthesis and is thus dependent on light and [CO_2_] conditions, as well as water availability, and mediated through signaling from the phytohormone abscisic acid (ABA) and the generation of reactive oxygen species (ROS) [13,14]. The influence of light on stomatal conductance varies by intensity and spectral quality: Low intensity blue-light initiates stomatal opening by activating a plasma membrane ATPase [15], while red light drives stomatal response via photosynthetic CO_2_ consumption [16]. CO_2_ also affects *G*_S_: Elevated [CO_2_] suppresses inward K+ channels and enhances outward K+ channels, depolarizing the guard cell membrane resulting in loss of turgor and stomatal closure [17]. Changes in stomatal aperture in response to [CO_2_] occur in response to intercellular [CO_2_] (C_i_) rather than atmospheric [CO_2_] [18]. C_i_ can oscillate between over 600 ppm in the dark to less than 200 ppm in the light [19]. Stomatal response to elevated [CO_2_] is mediated through ABA, with ABA increasing sensitivity to [CO_2_] [14]. For plants with a C3 photosynthetic pathway, elevated [CO_2_] means greater water use efficiency (WUE): more efficient photosynthesis and less water lost through transpiration (at least in the short term) [15]. Photosynthetic CO_2_ consumption is important, but the end products of photosynthesis, soluble sugars, also influence stomatal regulation.

The role of sugars in stomatal regulation is still under investigation. It was initially thought that sugar functioned as an osmolyte in stomatal movements, however, experiments with mannitol as an osmotic control showed that sucrose regulation of stomatal aperture was not an osmotic process [16] and sucrose actually induces stomatal closure at higher concentrations [17]. Sucrose accumulates in guard cells through photosynthesis, starch degradation, or apoplastic import from the mesophyll [18,19]. When biosynthesis of sucrose occurs in excess, sucrose is broken down into hexoses (glucose and fructose) by the sugar phosphorylating enzyme, hexokinase (HXK), which in turn initiates ABA-mediated stomatal closure [16]. This creates a natural feedback mechanism to limit transpiration when photosynthetic CO_2_ fixation is occurring in excess, causing an accumulation of sugars. HXK is also involved in the downregulation of photosynthesis under elevated [CO_2_] when plants lack sufficient sinks for the products of photosynthesis [15].

This study looks at how experimentally manipulated [CO_2_] and light conditions modulate both the stomatal density and stomatal conductance alongside physiological processes in two barley varieties differing in sensitivity to oxidative stress and phenolic compound profiles [20]—oxidative stress sensitive Barke and oxidative stress resistant Bojos. To measure stomatal density, we used a novel automated approach—a convolution neural network (CNN). The CNN was trained to recognize and count stomata on barley epidermal imprints. Here we also describe the process of training a CNN to count stomata on our dataset of micrographs. To better understand the biochemical and physiological background of stomatal regulation under changing light and [CO_2_] conditions we measured gas exchange parameters as well as the accumulation of sugars and ABA by biochemical assays.

Barley, as an economically important crop around the world, is increasingly exposed to conditions of limited water availability. Understanding of stomatal regulation under future CO_2_ conditions is thus vital to understanding and improving water use in barley. We hypothesized that low [CO_2_] and high light would increase stomatal density and WUE. We further hypothesized that the two barley varieties differing in their sensitivity to oxidative stress would show differences in their responsiveness to environmental driver in terms of stomatal density and conductance. Finally, we hypothesized that levels of hexose and ABA would provide insight into the regulation of stomatal conductance and contribute to our understanding of how stomata function as a key trait in plant response to environmental conditions. We provide a summary of how barley stomata are affected by light and [CO_2_] conditions and examine how pentoses, hexoses, disaccharides and ABA drive physiological changes in stomatal conductance.

## 2. Results

### 2.1. Changes in Stomatal Density

Stomatal densities varied significantly (*p* < 0.001) according to light treatment, [CO_2_] treatment, and barley genotype (Table 1). Low light (LL) conditions were set at 400 µmol m^−2^s^−1^ photosynthetically active radiation (PAR) with 0.75 W m^−2^ UV-A maxima and high light (HL) conditions were set at 1500 µmol m^−2^s^−1^ PAR with 4 W m^−2^ UV-A maxima. Low [CO_2_] (LC) conditions were set at 200 ppm, ambient [CO_2_] (AC) at 400 ppm, and elevated [CO_2_] (EC) at 700 ppm. For more details, see Material and Methods (Section 4.1). Stomatal density was measured on both the leaf abaxial and adaxial sides, however, no significant difference was found between sides in any treatments and so results show the average of stomata counted on both sides. Barley leaves grown in HL had significantly (*p* < 0.001) higher stomatal density than those grown in LL conditions (Table 2). Among the three [CO_2_] treatments, plants grown in LC had significantly (*p* < 0.001) higher stomatal densities than plants grown in either AC or EC, while there was not a significant difference between AC and EC leaves (Table 2).

**Figure 1:**
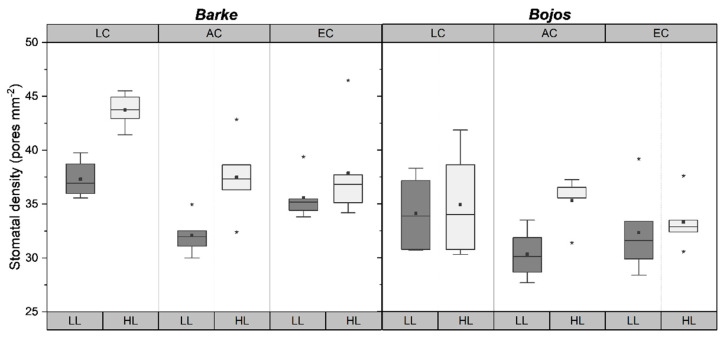
Boxplots showing differences in stomatal density mm^−2^ for barley genotypes, Barke (**left**) and Bojos (**right**). LC = low [CO_2_], AC = ambient [CO_2_], EC = elevated [CO_2_]; LL = low light (dark grey boxes), HL = high light (light grey boxes). Medians (central line), means (black squares), 25 and 75 percentiles (boxes), 1.5 interquartile range (error bars) and outliers (stars) are presented (*n* = 20).

Between the two barley genotypes, Barke plants had significantly (*p* < 0.001) higher stomatal densities than Bojos (Table 1 and Table 2). Both Barke and Bojos had significantly higher stomatal densities in high light conditions, however, this effect was more pronounced in Barke (*p* < 0.001) than it was for Bojos (*p* = 0.004) (Figure 1). The average HL density for Bojos was the same as the average LL density for Barke—35 per mm^2^ (Table 2). [CO_2_] significantly influenced stomatal density for Barke (*p* < 0.001) but not for Bojos (*p* = 0.088) (Figure 1).

### 2.2. Stomatal Function

**Figure 2:**
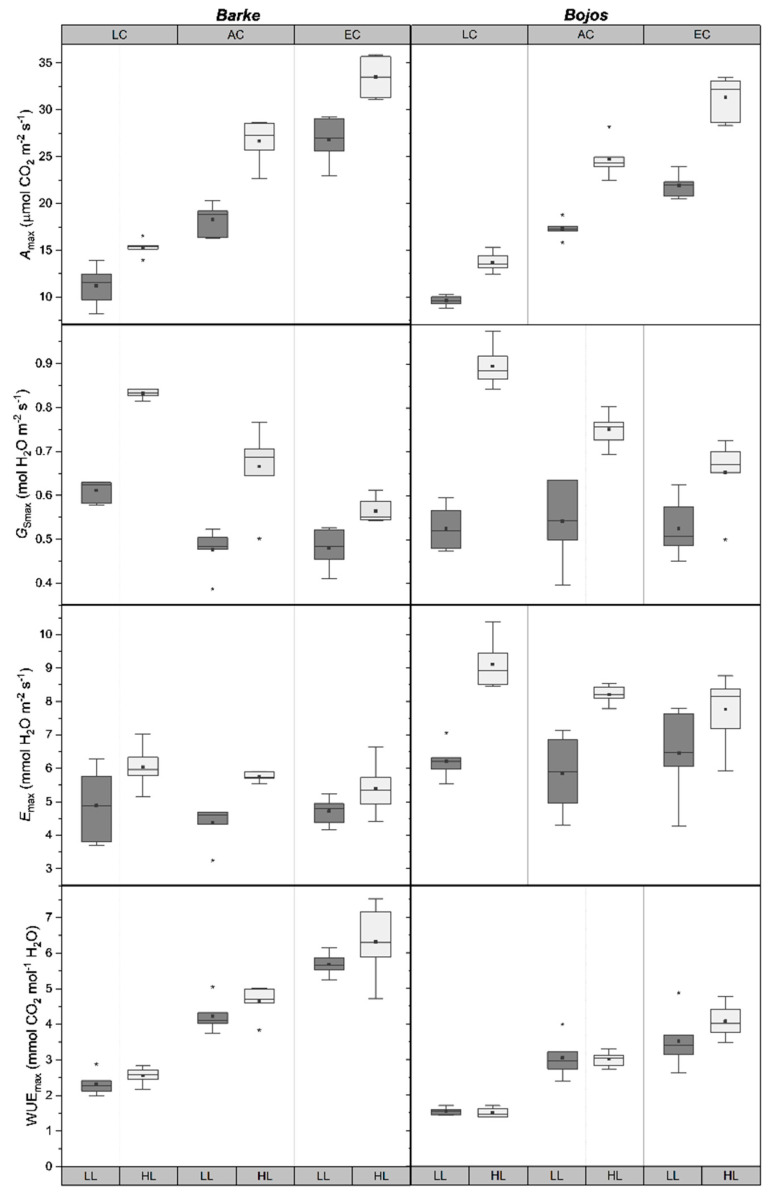
Boxplots showing differences gas exchange parameters for barley genotypes Barke (**left**) and Bojos (**right**). LC = low [CO_2_], AC = ambient [CO_2_], EC = elevated [CO_2_]; LL = low light (dark grey boxes), HL = high light (light grey boxes). Medians (central line), means (black squares), 25 and 75 percentiles (boxes), 1.5 interquartile range (error bars) and outliers (stars) are presented (*n* = 6).

Light, [CO_2_], and barley genotype all significantly (*p* ≤ 0.05) influenced transpiration (*E_max_*), photosynthesis (*A_max_*), stomatal conductance (*G_Smax_*), and WUE_max_, when measured at saturating light intensity (Table 1). HL plants had significantly (*p* < 0.001) higher levels of *E_max_*, *A_max_*, and *G_Smax_*, and higher WUE_max_ (*p* = 0.013) (Figure 2). LC plants had higher *E_max_* and *G_Smax_* and lower *A_max_* compared to AC and EC plants (Figure 2). In all cases, the highest rate of *G_Smax_* occurred for LC/HL plants, considerably higher than any other treatment combination (Figure 2). HL *G_Smax_* values were higher than under LL in all cases and decreased with increasing [CO_2_]. The differences between LL and HL *G_Smax_* also decreased with increasing [CO_2_] (Figure 2). WUE_max_ was significantly (*p* < 0.001) different between all three [CO_2_] treatments, with LC plants exhibiting the lowest WUE_max_ and EC plants exhibiting the highest WUE_max_ (Figure 2).

Differences between the stomatal behavior of the two barley genotypes were apparent. Bojos plants had significantly higher (*p* < 0.002) rates of *G_Smax_* and *E_max_*, and a lower WUE_max_ than Barke plants (Table 1, Figure 2). Not only did Barke show reduced transpiration compared to Bojos, but *E_max_* levels under LL for Bojos exceeded HL levels in Barke, and Bojos showed greater changes in *E_max_* between light treatments compared to Barke (Figure 2). WUE_max_ was significantly (*p* < 0.001) higher in Barke (Table 1), and the increase in WUE_max_ between each [CO_2_] treatment was greater between Barke plants compared to Bojos (Figure 2).

### 2.3. Sugar Metabolites

**Figure 3:**
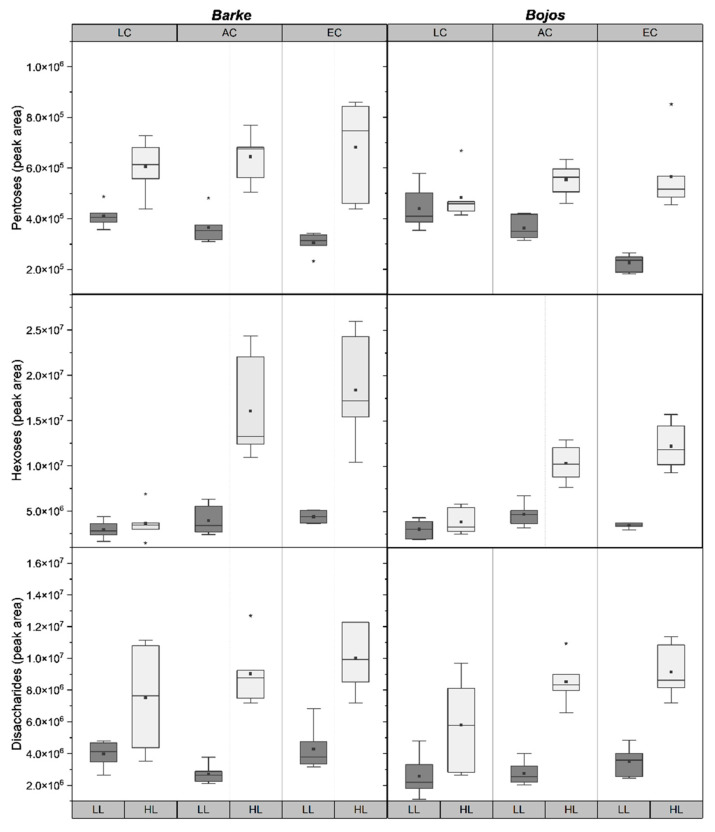
Boxplots showing differences in peak area sugars measured in barley genotypes, Barke (**left**) and Bojos (**right**). LC = low [CO_2_], AC = ambient [CO_2_], EC = elevated [CO_2_]; LL = low light (dark grey boxes), HL = high light (light grey boxes). Medians (central line), means (black squares), 25 and 75 percentiles (boxes), 1.5 interquartile range (error bars) and outliers (stars) are presented (*n* = 6).

Accumulation of sugars—pentoses, hexoses, and disaccharides—were all found to be significantly (*p* < 0.001) influenced by light conditions (Table 1). All HL plants had a greater accumulation of all sugars compared to LL plants, except for hexoses in LC, which did not show a difference between HL and LL (Figure 3). Genotype significantly influenced pentose (*p* = 0.005) and hexose (*p* = 0.003) levels, with Barke having higher levels of accumulation of both, and a greater increase at HL compared to Bojos (Figure 3). Hexose levels peaked for AC-HL and EC-HL plants for both genotypes (Figure 3). [CO_2_] significantly influenced hexose (*p* < 0.001) and disaccharides (*p* = 0.007) (Table 1). Disaccharides show an increasing trend alongside increasing [CO_2_] in HL but were relatively stable across [CO_2_] treatments in LL (Figure 3). Pentose was significantly influenced by a combination of genotype and light (*p* = 0.041) and [CO_2_] and light (*p* < 0.001): In both Barke and Bojos, levels of pentoses showed a slightly increasing trend with increasing [CO_2_] under HL, but a decreasing trend with increasing [CO_2_] under LL. The increase of pentoses under HL was more prominent for Barke, while the low light decrease was more prominent for Bojos (Figure 3).

### 2.4. Abscisic Acid

**Figure 4:**
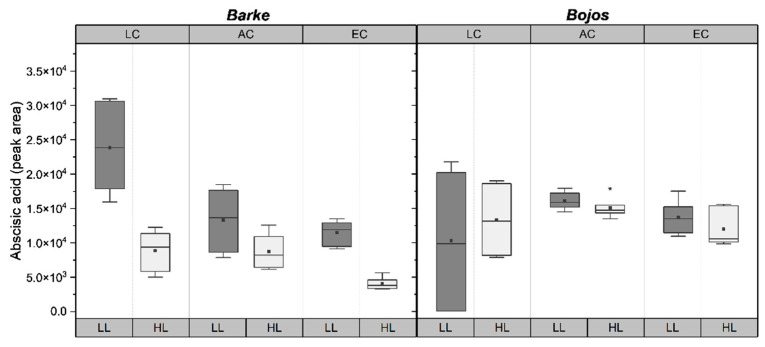
Boxplots showing differences in peak area abscisic acid measured in barley genotypes, Barke (**left**) and Bojos (**right**). LC = low [CO_2_], AC = ambient [CO_2_], EC = elevated [CO_2_]; LL = low light (dark grey boxes), HL = high light (light grey boxes). Medians (central line), means (black squares), 25 and 75 percentiles (boxes), 1.5 interquartile range (error bars), and outliers (stars) are presented (*n* = 6).

Peak area of ABA was influenced by almost all tested environmental factors and was the only measured parameter to show significant differences driven by the interactions of genotype, [CO_2_], and light in combination (Table 1). Generally, ABA decreased in EC and HL. LC-LL plants had the highest ABA levels, while EC-HL plants had the lowest ABA levels (Figure 4). Barke plants showed higher variability between HL and LL, as well as higher variability among [CO_2_] treatments compared to Bojos genotype (Figure 4).

### 2.5. Redundancy Analysis

**Figure 5:**
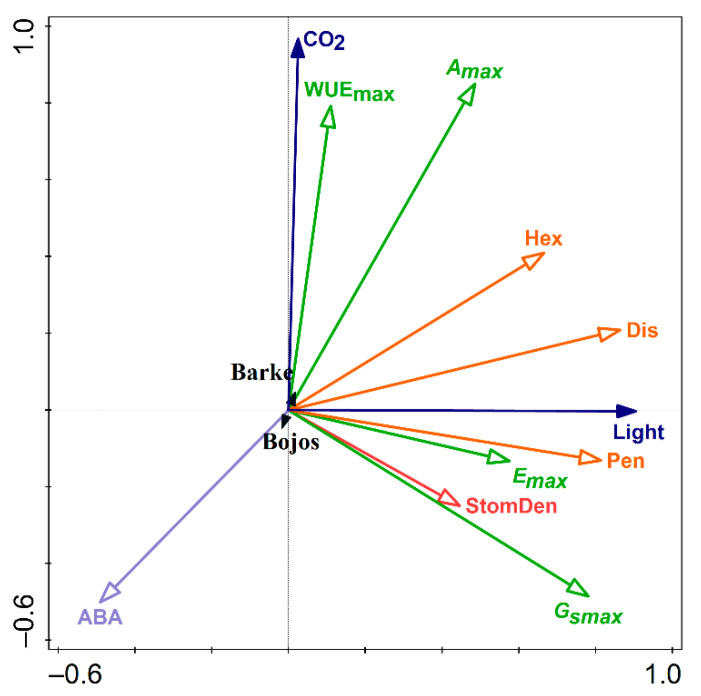
Biplot diagram representing the results of redundancy analysis (RDA) on the effects of light intensity (Light), CO_2_ concentration (CO_2_), and genotype (Barke and Bojos) on stomatal density (StomDen), accumulation of pentose (Pen), disaccharides (Dis), hexose (Hex), and abscisic acid (ABA), and parameters of stomatal function: Photosynthesis (*A_max_*), Transpiration (*E_max_*), stomatal conductance (*G_Smax_*), and water use efficiency (WUE_max_).

Associations between environmental drivers (light intensity, [CO_2_]), genotype (Barke, Bojos) and anatomical, physiological, and biochemical parameters related to stomatal function were tested using redundancy analysis (RDA). The explained cumulative variation by components 1 and 2 was 83.23%, pseudo-F = 34.2, *p* = 0.002. [CO_2_] was most positively correlated with WUE and *A_max_* (Figure 5). Accumulation of sugars, especially disaccharides and pentoses, positively associated more closely with light, although hexoses were nearly split between the influence of light and the influence of [CO_2_] (Figure 5). *G_Smax_* and stomatal density correlated positively, but stomatal density did not explain the full extent of *G_Smax_*, which was additionally negatively related to ABA (Figure 5). The interactive effect of [CO_2_] and light was most pronounced positively on *A_max_* and accumulation of hexoses, and negatively on accumulation of ABA. This resulted in negative associations between ABA and *A_max_,* and between ABA and hexoses. Pentose levels and *E_max_* also correlated (Figure 5). WUE_max_, *A_max_*, and the effect of [CO_2_] were more associated with Barke than Bojos, while ABA and *G_Smax_* levels were more associated with the Bojos genotype, however, the effect of barley genotype was rather small (Figure 5).

## 3. Discussion

Considerable attention has been given to stomata as a target for improving the resilience of crops to reduced water availability in the face of ongoing climate change. Changes in stomatal density have implications for crop productivity. For instance, manipulation of the EPF (epidermal patterning factor) family of signaling peptides to reduce stomatal density in barley may improve drought tolerance [21,22]. However, a reduction in stomatal density may lead to more residual transpiration and potential decreases in tolerance to stress factors, such as salinity [23]. Changes in stomatal density affect WUE [24] and this trait can be used to improve WUE through genetic manipulation [25]. Thus, stomatal density plays a role in determining plant productivity, especially in the context of environmental stress, particularly drought.

**Figure 6:**
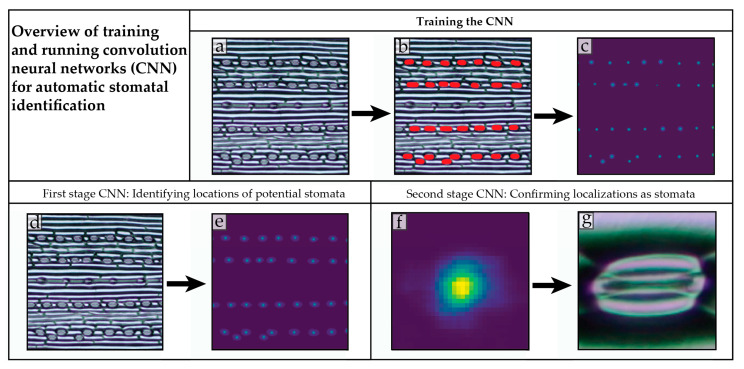
Basic outline of how the convolution neural network (CNN) was trained, and how it functioned in two stages to determine stomatal density. To train the CNN, first unmarked micrographs (**a**) were manually labelled, highlighting stomata in red (**b**) to indicate to the CNN what feature to search for. The output of the training was a label image generated by the CNN (**c**). The label image consisted of Gaussian peaks centered on stomata which enabled the network to extract relevant 2D features for the following two stages. Once the CNN was trained, the first stage CNN scanned an unmarked epidermal micrograph (**d**) for possible stomatal complexes and output a heatmap of potential locations (**e**). The second stage CNN was centered on windows around each heatmap reaction point from the previous CNN (**f**) and determined if the visual object is a stomatal complex or not (**g**).

Manual counting of stomatal cells can be a tedious and time-consuming task, especially when large datasets are involved. The idea of using computer algorithms to count stomata has received some attention in recent years. Recently a CNN was developed to recognize stomata across species and was made publicly available [26]. However, we found that a CNN created specifically for our data set worked faster and more reliably. Moreover, our case-tailored CNN could be trained to ignore human-generated defects in the images, such as air bubbles captured on slides. By using a CNN trained on our dataset, we were able to achieve results comparable to human counting and maintain the ability to reuse and even retrain our CNN to fit future research questions (Figure 6).

Although the effects of elevated [CO_2_] and light intensity on stomatal density and stomatal conductance have been previously described separately [7,27], the interactive effects of [CO_2_] and light on stomata regulation are not yet sufficiently understood. Research on *Arabidopsis* suggests that the light and CO_2_ signaling pathways for stomatal opening are linked [28]. To our knowledge, there are only two existing studies of how the stomatal density of barley specifically is affected by light conditions [29,30], and no data on how stomatal density is affected by [CO_2_] in barley varieties not specifically genetically modified for stomatal density [21]. In this study, we aimed to improve understanding of the interwoven relationships between stomatal density, stomatal conductance, sugar metabolism, and ABA in relation to changing [CO_2_] and light intensity and the possible impacts on WUE. Additional graphs representing the effect of light and [CO_2_] on barley for all measured parameters independent of genotype can be found in in the supplementary material (Figure S1).

High light intensity is known to increase stomatal density [31,32]. The molecular background for the increase in stomatal density under HL conditions has been identified as signaling peptide known as STOMAGEN or EPFL9, which encodes an epidermal patterning factor that positively influences stomatal density and index on both sides of the leaf [33,34]. A previous study on the effect of light on stomatal density in barley showed no significant difference between leaves grown in LL versus HL environments [30], however that study used significantly lower levels of light intensity even for HL environment (200 µmol m^−2^s^−1^), which is half of the intensity of our LL conditions. Our high light treatment conditions coincided with the average light saturation point for barley (1500 µmol m^−2^s^−1^) [35] and shows a strong positive effect on stomatal density (Table 1, Figure 1). This suggests that light can increase the stomatal density at high intensities, however, perhaps stomatal density will not be decreased by progressively dimmer light below a certain point.

While specific data regarding the effect of [CO_2_] on stomata in barley are missing, there are several studies on the effect of altered [CO_2_] on stomata in wheat, *Triticum aestivum* [8,36,37]. A study on the influence of [CO_2_] in wheat found that stomatal density decreased significantly with increasing [CO_2_] [38]. Studies on *Arabidopsis* have identified the HIC (high carbon dioxide) gene, which encodes an enzyme involved in the negative regulation of stomatal development in elevated [CO_2_] [39]. Our results show that barley had a higher stomatal density at LC, but differences between AC and EC were not pronounced (Figure 1). This discrepancy may be because the study on wheat [38] used a [CO_2_] range between 400 and 1200 ppm, so perhaps the difference between 400 and 700 ppm in this study was not great enough to elicit a strong significant response in terms of stomatal density. However, in the context of climate change, 700 ppm is a potential natural atmospheric [CO_2_] for the end of the 21st century [40], while values above that are unlikely to be encountered by crop plants. Furthermore, an older study on wheat also did not find significant differences between stomatal density at ambient (370 ppm) vs. elevated (550 ppm), indicating that EC increase in stomatal density occurs only at sufficiently higher atmospheric [CO_2_] (i.e., above 700 ppm). Investigation of the low [CO_2_] response was included in this study to address past ecophysiological function. The 200-ppm value used for LC plants is representative of atmospheric CO_2_ levels experienced by plants during the last glacial period [41,42]. The influence of both low and elevated [CO_2_] was more pronounced for Barke than for Bojos (Figure 1), which is not surprising as barley displays great genetic diversity and a recent report on wheat showed significant genetic variation in terms of stomatal response [43].

Past studies have generally found a positive relationship between stomatal density and *G_Smax_* [23,24,44]. *Arabidopsis* mutants with reduced stomatal densities have lower *G_Smax_* and increase WUE by up to 20% [25]. Our results did show higher stomatal densities (Figure 1) corresponded with higher *G_Smax_* (Figure 2) between individual treatments. RDA analysis shows that stomatal density and *G_Smax_* did positively correlate, however that stomatal density did not fully explain the full extent of the measured *G_Smax_* (Figure 5). Our results also showed that the genotype with a greater stomatal density, Barke, had a greater WUE_max_ than the genotype with a lower stomatal density, Bojos (Figure 2). This indicates the possibility of compensating mechanisms, such as more sensitive stomatal aperture regulation, as being a greater driver of WUE_max_ than stomatal density among barley genotypes. In fact, the RDA graph suggests a slight correlation of *G_Smax_* with ABA (Figure 5). The compensation of altered stomatal density by regulating stomatal aperture was previously described in *Arabidopsis* stomatal mutants and the mechanism appeared to be light-dependent [45]. Other studies on crop grass species have noted a decoupling of stomatal density with *G_Smax_*. For instance, one study on wheat cultivars found significant differences between stomatal densities, as well as *A_max_*, *E_max_*, and WUE_max_, despite no significant differences in *G_Smax_* levels [46]. In another study, overexpression of the maize gene SHORTROOT1 (ZmSHR1) in rice led to increased stomatal density, but no corresponding change *G_Smax_* [47]. Previous studies also indicate interactions between light intensity and [CO_2_] play a role in *G_Smax_* regulation. Studies on *Fagus sylvatica* showed the stomata of EC plants remained closed at light intensity up to 500 µmol m^−2^s^−1^, suggesting that plants grown in EC may require a higher activation energy to trigger stomatal opening [48]. Low intensity light may lack sufficient energy to activate the biochemical processes for stomatal opening when [CO_2_] is high. Our results show a positive effect of [CO_2_] on WUE_max_, which is typical of stomatal closure under EC conditions (Figure 5).

Differences in accumulation of metabolites may account for the differences in stomatal behavior between genotypes [49,50]. The energy required to drive stomatal regulation exceeds the synthetic capacity of guard cells, meaning mesophyll cell support guard cell by providing metabolites and ATP [51,52]. Hexoses enter the guard cells and induce stomatal closure via a hexokinase mediated mechanism mediated by ABA [16]. Barke did show higher hexose levels—although mainly for AC-HL and EC-HL (Figure 3). Hexoses were negatively correlated with ABA in the RDA graph (Figure 5). Moreover, the genotypes Barke and Bojos were previously characterized in our recent work on the accumulation of phenolic compounds in barley [20]. We found that Barke, considered an oxidative stress sensitive genotype, accumulated more hydroxybenzoic acids, while Bojos, an oxidative stress resistant genotype, accumulated more hydroxycinnamic acids. For both species, soluble phenolics were not identified in the epidermis, except in stomatal guard cells. The accumulation of phenolic compounds in guard cells but not pavements cells has also been documented for *Arabidopsis*, where their presence in the guard cells was hypothesized to play a role in ABA signaling and stomatal aperture due to their antioxidative properties [53]. ABA acts in stomata regulation with the help of reactive oxygen species (ROS). ROS (hydrogen peroxide, H_2_O_2_; superoxide, O_2_^−^; hydroxyl radical, OH^−^; and singlet oxygen, ^1^O_2_) are known to cause oxidative damage to lipid membranes, proteins, and DNA, but H_2_O_2_ in particular plays an integral role as secondary messengers in signal transduction pathways [54]. ABA triggers the production of H_2_O_2_ by activating respiratory burst oxidase homolog enzymes on the plasma membrane [55]. This ROS burst triggers calcium channels to increase cytosolic Ca^2+^ and activate anion efflux channels resulting in stomatal closure [56,57]. For a recent review of ABA effects in crop grasses, see Gietler et al. [58]. Our results show lower levels of ABA (except for treatment at LC-LL) (Figure 4) as well as lower transpiration and higher WUE_max_ (Figure 2) for Barke, the genotype known to accumulate fewer hydroxycinnamic acids and be more sensitive to oxidative stress. The structure of hydroxycinnamic acids makes them more efficient at scavenging ROS [59]. The comparative lack of hydroxycinnamic acids in Barke may make ABA-induced ROS signaling more efficient, thus requiring lower ABA levels to trigger stomatal closure. In fact, studies on *Arabidopsis* indicated that mutants lacking phenolic compounds in their guard cells were more sensitive to ABA-induced ROS signaling. Phenolic compounds in guard cells may be scavenging ROS to a high enough degree that they modulate ABA-signaling and stomatal aperture [53]. Although no studies have thus far been conducted on barley, a study on *Commelina communis* found that p-coumaric, caffeic, chlorogenic, salicylic, and sinapic acids inhibited stomatal opening, while ferulic acid stimulated stomatal opening [60]. Another study on *Lactuca sativa* showed a commensurate increase in *G_Smax_* alongside blue-light induced accumulation of phenolic acids and flavonoids [61]. There is still a paucity of research exploring the relationship between ABA-dependent stomatal signaling and phenolic compounds, however it remains an intriguing question for future investigation.

Physiological studies have demonstrated convergent and antagonistic effect of ABA and sugars on stomatal response [62]. As sucrose accumulates in the apoplast, water moves out of guard cells osmotically, creating an inverse relationship between photosynthetic rate and transpiration [63,64]. Water soluble carbohydrates, such as sucrose, glucose, and fructose, serve as indicators of assimilate accumulation. Metabolism of sucrose into hexoses provides substrate for glycolysis and biosynthesis of other essential molecules, such as starch, cellulose, and fructan [65]. ABA influences overall plant metabolism by inducing the conversion of sucrose into hexose by cell wall invertase (CWIN) which is taken into the cytoplasm and enhances sink strength [65]. Levels of hexoses are sensed by nucleus-located hexokinase (HXK) which phosphorylates glucose, plays a role in sugar sensing, and regulates gene expression [65,66]. Our results show that sugar accumulation was higher in all HL plants (Figure 3), while ABA levels were lower in HL plants (Figure 4). Studies on rice (*Oryza sativa*) show that sugar starvation increases concentrations of ABA and ROS, inducing programmed cell death and early senescence [67]. In tomato plants overexpressing HXK, ABA concentrations spiked and induced early senescence [68]. Increased ABA levels promote H_2_O_2_ production which, in turn, acts as a signal to reduce stomatal aperture [52]. This corresponds with our RDA, indicating a negative relationship between hexoses and ABA, and a slight influence of ABA on *G_Smax_* (Figure 5).

## 4. Materials and Methods

### 4.1. Plant Material

Barley plants (*Hordeum vulgare* L.) of either Barke (sensitive to oxidative stress [69,70]), or Bojos (relatively resistant to oxidative stress [71]) varieties were grown in growth chambers (FS-SI-3400, Photon System Instruments, Drásov, CZ). Cultivation occurred over four weeks under three different [CO_2_] treatments: low [CO_2_]—200 ppm (LC), ambient [CO_2_]—400 ppm (AC), and elevated [CO_2_]—700 ppm (EC) and two light regimes: low light (LL) with photosynthetically active radiation (PAR) and UV-A maxima of 400 µmol m^−2^s^−1^ and 0.75 W m^−2^, respectively and high light (HL) with PAR and UV-A maxima 1500 µmol m^−2^s^−1^ and 4 W m^−2^, respectively. The light intensity, temperature, and air humidity changed gradually to simulate natural rhythms with 15 h:9 h, light:dark. Air temperature and relative air humidity varied between 15–25 °C and 90–60%, respectively.

After 4 weeks, the third leaf from the top was sampled. Stomatal imprints were made using clear nail varnish on the middle of a fresh leaf. The nail varnish was applied to the leaf in a thin layer, and then peeled off when sufficiently dry and taped to a microscope slide. These stomatal imprints were made on both the abaxial and adaxial sides of the leaves. Stomatal imprints were photographed at 10× magnification using an Olympus BX40 light microscope equipped with Canon EOS100D camera.

### 4.2. Training a Convolution Neural Network to Recognize Stomata

To streamline the process of counting stomata, a convolution neural network (CNN) was trained to recognize and count stomata. A CNN is a type of neural network particularly efficient at recognizing two-dimensional patterns and extracting searchable features from pixel images [72]. Our stomata counting program was written in the Python programming language. The TensorFlow framework and Keras [73] library was used to develop the CNN, and a human-in-the-loop approach helped to quickly extend the training set and maximize performance. The complete model combines two CNNs working in distinct processing phases. The first CNN takes a micrograph of barley leaf epidermis and outputs a heatmap of predicted stomatal locations. The second CNN takes cropped windows of the predicted stomatal locations and classifies the window as either containing a stomatal complex, or not.

In the initial training phase, representative micrographs of barley epidermal imprints were labelled: Labelled images were generated by using source microscopy image and highlighting the cells with red color (#FF0000) using the brush tool in a graphics editor and leaving the rest of the epidermis unmarked. The input images were originally sized at 3456 × 5184, but due to hardware limitations, were cut into 6 tile-pairs for processing.

The red-colored areas corresponding to stomata locations in the training images were extracted as a binary matrix the size of the image: 0 for pixels that do not contain stomata and 1 for pixels that do. The labelled stomatal areas were replaced by gaussian peaks centered on the stomatal complex (a two-dimensional gaussian function). This removed the discontinuity of the label function on the edge of the cell, making it continuous, and making detection of the stomatal complex easier. This generated 1396 training image pairs and 156 testing/validation images (12 tiles for each original image, pairs consist of one original micrograph tile and one label-image tile. Of the total training image dataset, 90% of labeled image-pairs were allocated to training and 10% were used for testing.

The first CNN consisted of six convolutional layers interspersed with normalization layers (Batch normalization [74] and Dropout [75]), and two Max Pooling layers that reduced the size of the image by a factor of four. The first two convolution layers utilized ReLu activation [76], and the next four utilized sigmoid activation. To extract cell centers, images are blurred using a gaussian blur and thresholded. Afterwards, an algorithm extracted the cell complex centers.

The second stage CNN was a classifier with a binary output. Output from the first stage became input for the second stage. The training data was extracted from the training images processed by the first stage model. Input of the second stage CNN was a 32 × 32-pixel window centered on predicted cell locations from the first stage CNN heatmap output. Output of the second CNN was a single number from 0 to 1, representing the estimated probability of the window containing a stomata cell in the center. The second model is composed of 2 convolution layers with a ReLu activation function and batch normalization, and 2 fully connected layers with sigmoid activation function. See Figure 6 for a basic overview, and Supplementary Material (Figures S2 and S3) for the full model of both stages.

To ensure unbiased sampling, stereological principles were incorporated after the CNN was applied—specifically, an unbiased counting frame. The idea behind a counting frame is that, if the whole leaf blade was sampled by counting frames lying next to each other but not overlapping, each object (in this case, stomatal complex) would have the same probability to be sampled, i.e., would only be counted once, even if appearing in two counting frames [77]. This is achieved by having 2 borders (usually top and right sides) as inclusionary, meaning that stomata appearing partially on these borders are counted. Conversely, stomata appearing on the exclusionary borders (bottom and left sides) are not counted. Exclusionary borders then continue behind the borders of counting frame—left side continuing to the top and right side continuing to the bottom (see Figure S4 for visualization). All stomata that are fully inside the borders are counted [77,78,79]. To achieve this effect, boundaries were defined in terms of pixels from the edge of the image. Stomatal complex centers detected lying on the boundary lines were counted according to whether they fell on an inclusionary or exclusionary boundary. Stomatal frequency can be measured in terms of stomatal density (SD): the number of stomata per unit leaf area, or as stomatal index, SI: the number of stomata relative to the total number of epidermal cells [80]. We use stomatal density rather than stomatal index because the epidermal cells of grass are significantly longer than stomata and extend past the borders of our counting frame.

To test the efficacy of the CNN in correctly identifying and counting stomata, four identification scenarios had to be considered: (1) True positive ($N_{TP}$), correct labeling of a stomata, (2) False positive ($N_{FP}$), labeling a non-stomata as a stomata, (3) True negative ($N_{TN}$), non-labelling of a non-stomata, and (4) False negative ($N_{FN})$, non-labeling of a stomata. From these parameters, it was possible to assess the accuracy, precision, and recall of the CNN. Precision (P) was defined as: $P=\frac{N_{TP}}{N_{TP}+N_{FP}}$. Recall (R) was defined as: $R=\frac{N_{TP}}{N_{TP}+N_{FN}}$. Accuracy (*A*c) was defined as: $Ac=\frac{N_{TP}+N_{FN}}{N_{TP}+N_{FP}+N_{TN}+N_{FN}}$.

The first stage CNN generated 1332 true positives, 25 false positives, and 55 false negatives, resulting a precision of 98.1% and a recall of 96% after 68 epochs. (An epoch is running all the training images through the algorithm once).

The dataset for the second stage CNN model (generated from the training set of the first model) consisted of 24,033 potential cell-location windows: 8881 of the potential cell windows contained stomata and 15,152 of the potential cell windows were merely artifacts (i.e., small bubbles, thorn cells, or imprint damages) which created a detection response (hotspot) in the heatmap. The accuracy score was 98.5%.

### 4.3. Gas Exchange Measurements

Leaf level gas exchange measurements were performed on intact leaves of light adapted plants between 10:00–15:00 CET. Light saturated (1200 μmol photons m^−2^s^−1^) CO_2_ assimilation rate (*A_max_*), stomatal conductance (*G*_Smax_), transpiration rate (*E_max_*) were measured using open path gas exchange system Li-6800 (LiCOR, LI-COR Biosciences, Lincoln, NE, USA) at growth [CO_2_] (200, 400, and 700 ppm for LC, AC, and EC respectively), relative air humidity of 60%, and air temperature of 25 °C. Water use efficiency (WUE) was then calculated as the ratio between *A*_max_ and *E_max_* at saturating light intensity.

### 4.4. Identification of Sugar Metabolites and Phytohormones

Approximately 0.3 g of leaves for metabolite analyses were sampled between 10:00 and 15:00 CET directly after gas exchange measurement, immediately placed into liquid nitrogen, and then stored at 80 °C until the time of processing. Levels of barley metabolites were measured in terms of peak area using liquid chromatography coupled with mass spectrometry according to the protocol in Večerová et al., 2019 [81]. The samples were homogenized and extracted in a methanol:chloroform:H_2_O solution (1:2:2). An aliquot of the upper (polar) phase was used to identify pentoses, hexoses, and disaccharides. An UltiMate 3000 high performance liquid chromatograph (HPLC) coupled with an LTQ Orbitrap XL high-resolution mass spectrometer (HRMS) (Thermo Fisher Scientific, Waltham, MA, USA) was used. A Hypersil GOLD column (150 × 2.1 mm, 3 mm; Thermo Fisher Scientific) temperated at 30 °C was used for separation. The flow rate of the mobile phase (acetonitrile and water with 0.1% acetic acid) was 0.3 mL min^–1^. The HRMS was equipped with a HESI II heated electrospray ionization source (Thermo Fisher Scientific, Waltham, MA, USA) and was operated at the full scan resolution of 60,000. Full scan spectra were acquired over the mass range of 50–1000 and 65–1000 *m*/*z* in positive and negative polarity mode, respectively. The target compounds were assigned based on our own mass library created using standards measured in MS and MS^n^ modes.

### 4.5. Statistical Analysis

Three-way analysis of variance (ANOVA) was used for the analysis of barley genotype, [CO_2_] and light intensity effects and their interactions. Fisher’s LSD ANOVA post-hoc test (*p* = 0.05) was used to analyze significant differences between means. Statistical analyses were conducted using the software STATISTICA 12 (StatSoft, Tulsa, CA, USA). The bar graphs representing means with standard errors were developed with the software SigmaPlot 11.0 (Systat Software, San Jose, CA, USA). The redundancy analysis (RDA) and biplot of RDA results were set up in the software CANOCO 5 (Microcomputer Power, Ithaca, NY, USA) [82].

## 5. Conclusions

In conclusion, although barley varieties show significant differences in stomatal density, and especially high differences in responsiveness of stomatal density to [CO_2_] and light conditions, levels of *G_Smax_* are similar for both genotypes, indicating some mechanisms for compensating the stomatal density differences to optimize the stomatal conductance to a given environment. Such mechanisms could be associated with differences in accumulation of hexoses and responsiveness of ABA to [CO_2_] and light conditions, which were generally higher in genotype Barke. Such differences could also explain the generally higher WUE in genotype Barke, although higher WUE is often reported as trait linked with lower stomatal density (which was the case for the Bojos genotype). Barke is a genotype known to be more sensitive to oxidative stress and have fewer hydroxycinnamic acids, which may make it more sensitive to the ROS initiated ABA signaling cascades. Crop plants, such as barley, are already facing harsher and more erratic environmental conditions due to ongoing climate change. Investigations on genotype-specific responses of important grain crops expand the availability of useful information which can lead to selection or modification of more resistant crop genotypes.

## Figures and Tables

**Table 1:** Results of a three-way ANOVA test showing interaction between experimental factors. Statistical significance is denoted by bold font. Significance was established at *p* ≤ 0.05. Abbreviations are defined as *Emax* (maximum transpiration rate), *Amax* (maximum photosynthesis rate), *G_Smax_* (maximum stomatal conductance), WUE_max_ (maximum water use efficiency), and ABA (abscisic acid).

Treatment	Stomatal Density	*E_max_*	*A_max_*	*Gs_max_*	WUE_max_	Pentoses	Hexoses	Disacch-Arides	ABA
**Genotype (G)**	**<0.001**	**<0.001**	**<0.001**	**0.002**	**<0.001**	**0.005**	**0.003**	0.054	**0.002**
**CO_2_**	**<0.001**	**0.046**	**<0.001**	**<0.001**	**<0.001**	0.268	**<0.001**	**0.007**	**<0.001**
**Light (L)**	**<0.001**	**<0.001**	**<0.001**	**<0.001**	**0.013**	**<0.001**	**<0.001**	**<0.001**	**<0.001**
**G × CO_2_**	0.068	0.869	0.073	**0.018**	**<0.001**	0.550	0.060	0.474	0.272
**G × L**	0.084	**0.004**	0.472	**0.010**	0.253	**0.041**	**0.004**	0.715	**<0.001**
**CO_2_ × L**	0.119	0.059	**<0.001**	**<0.001**	0.171	**<0.001**	**<0.001**	**0.037**	**0.017**
**G × CO_2_ × L**	0.273	0.464	0.166	0.120	0.802	0.576	0.091	0.980	**0.021**

**Table 2:** Average stomatal density mm^−2^ for two barley genotypes, Barke and Bojos, in various experimental groups ± standard deviation. LC = low [CO_2_], AC = ambient [CO_2_], EC = elevated [CO_2_]; HL = high light, LL = low light.

Stomata Density (Pores mm^−2^)					
All Genotypes		Barke		Bojos	
Average	35 ± 7	Average	37 ± 8	Average	33 ± 7
HL	37 ± 8	HL	40 ± 8	HL	35 ± 7
LL	33 ± 7	LL	35 ± 7	LL	32 ± 6
LC	37 ± 8	LC	40 ± 8	LC	35 ± 6
AC	34 ± 7	AC	35 ± 7	AC	33 ± 7
EC	35 ± 7	EC	37 ± 7	EC	33 ± 6

## Data Availability

Data is contained within the article or supplementary materials.

## Supplementary Materials

The following are available online at https://www.mdpi.com/article/10.3390/plants10112533/s1, Figure S1: Graphs of all measured parameters showing the effects of light and [CO_2_] without genotype, Figure S2: Model of first stage CNN, Figure S3: Model of second stage CNN, Figure S4: Stereological counting frame, Table S1: Full data set.
