# Supplementary material for: Barley Genotypes Vary in Stomatal Responsiveness to Light and CO2 Conditions
Source: Plants (Basel). 2021 Nov 21;10(11):2533. doi: 10.3390/plants10112533 (PMC8625854; doi:10.3390/plants10112533)
Supplement: Supplementary file 1 [file plants-10-02533-s001.zip › Supplementary files/Supplementary Figures S1-S4.pdf]

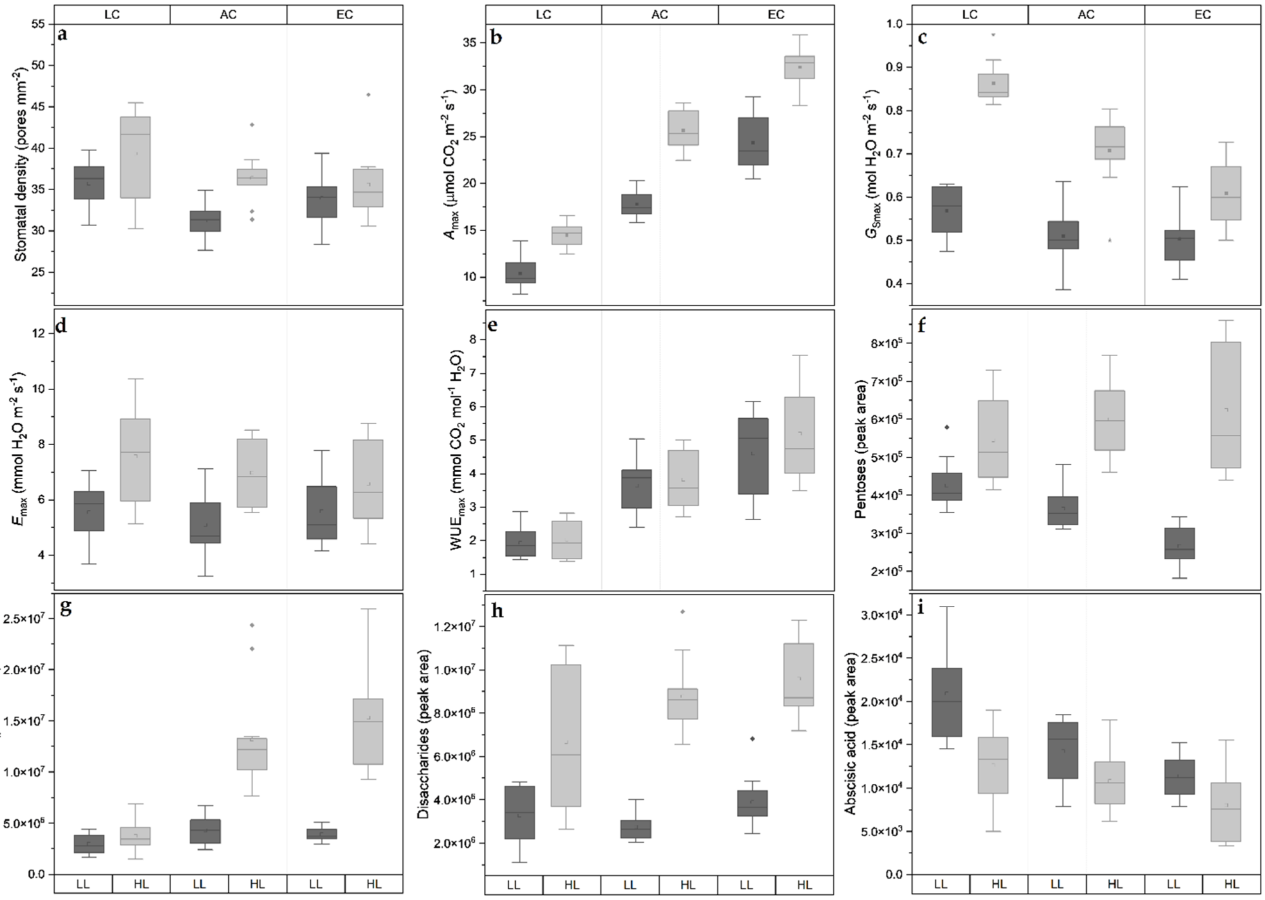

Figure S1. Boxplots showing the effect of light and [CO_2] on the studied parameters without the influence of genotype. Stomatal density in mm^{-2} (a); Gas exchange parameters: maximum photosynthesis (A_{max}) (b), maximum stomatal conductance (G_{Smax}) (c), maximum transpiration (E_{max}) (d), maximum water use efficiency (WUE_{max}) (e). Peak area sugars: pentoses (f), hexoses (g), disaccharides (h). Peak area abscisic acid (i). LC = low [CO_2], AC = ambient [CO_2], EC = elevated [CO_2]; HL = high light (light grey boxes), LL = low light (dark grey boxes). Medians (central line), means (black squares), 25 and 75 percentiles (boxes), 1.5 interquartile range (error bars) and outliers (stars) are presented.

Model: First stage CNN

Layer (type)	Output Shape	Param #
conv2d (Conv2D)	(None, None, None, 4)	112
activation (Activation)	(None, None, None, 4)	0
batch_normalization (BatchNo	(None, None, None, 4)	16
max_pooling2d (MaxPooling2D)	(None, None, None, 4)	0
dropout (Dropout)	(None, None, None, 4)	0
conv2d (Conv2D)	(None, None, None, 4)	404
Activation (Activation)	(None, None, None, 4)	0
batch_normalization (Batch	(None, None, None, 4)	16
max_pooling2d (MaxPooling2	(None, None, None, 4)	0
Dropout (Dropout)	(None, None, None, 4)	0
conv2d (Conv2D)	(None, None, None, 12)	3900
Activation (Activation)	(None, None, None, 12)	0
batch_normalization (Batch	(None, None, None, 12)	48
conv2d (Conv2D)	(None, None, None, 12)	11676
Activation (Activation)	(None, None, None, 12)	0
batch_normalization (Batch	(None, None, None, 12)	48
conv2d (Conv2D)	(None, None, None, 4)	3892
Activation (Activation)	(None, None, None, 4)	0
batch_normalization (Batch	(None, None, None, 4)	16
conv2d (Conv2D)	(None, None, None, 1)	101
Activation (Activation)	(None, None, None, 1)	0

Total params: 20,229
Trainable params: 20,157
Non-trainable params: 72

Figure S2. Model of the first stage CNN.

Model: Second stage CNN

Layer (type)	Output Shape	Param #
conv2d (Conv2D)	(None, 32, 32, 6)	1740
Activation (Activation)	(None, 32, 32, 6)	0
batch_normalization (Batc	(None, 32, 32, 6)	24
conv2d (Conv2D)	(None, 32, 32, 2)	3470
Activation (Activation)	(None, 32, 32, 2)	0
batch_normalization (Batc	(None, 32, 32, 2)	8
Flatten (Flatten)	(None, 2048)	0
Dense (Dense)	(None, 12)	24588
Dense (Dense)	(None, 1)	13

Total params: 29,843
Trainable params: 29,827
Non-trainable params: 16

Figure S3. Model of the second stage CNN.

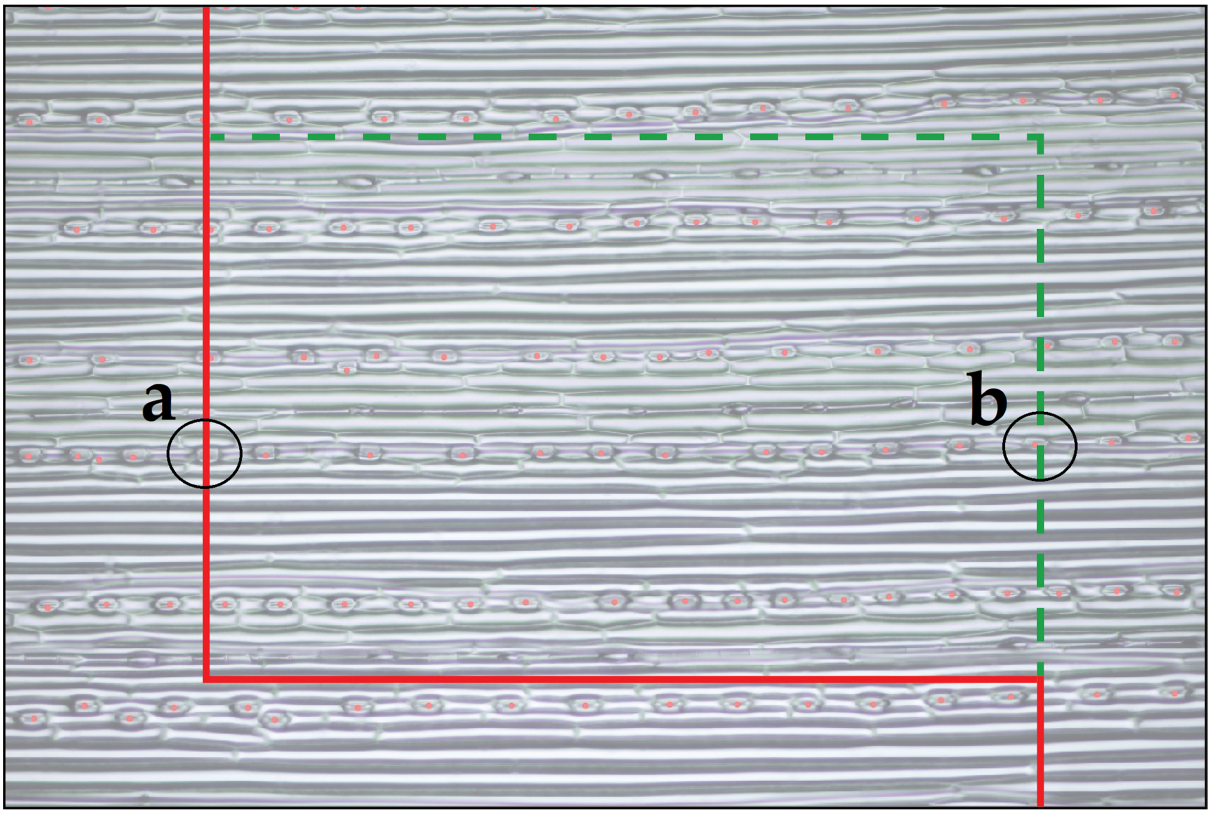

Figure S4. Stereological counting frame. The solid red line is the exclusionary border. Stomata that fall on the red line (a) will not be counted. The dashed green line is the inclusionary border. Stomata that fall on the green line (b) will be counted.
